# Identification of an attenuated barley stripe mosaic virus for the virus-induced gene silencing of pathogenesis-related wheat genes

**DOI:** 10.1186/s13007-016-0112-z

**Published:** 2016-02-02

**Authors:** Leann M. Buhrow, Shawn M. Clark, Michele C. Loewen

**Affiliations:** Aquatic and Crop Resources Development Portfolio, National Research Council of Canada, 110 Gymnasium Place, Saskatoon, SK S7N 0W9 Canada; Department of Biochemistry, University of Saskatchewan, 107 Wiggins Rd., Saskatoon, SK S7N 5E5 Canada

**Keywords:** Salicylic acid, Jasmonic acid, Abscisic acid, Pathogenesis-related 1, Deoxynivalenol, Vomitoxin, Yield, Necrotrophic fungi

## Abstract

**Background:**

Virus-induced gene silencing (VIGS) has become an emerging technology for the rapid, efficient functional genomic screening of monocot and dicot species. The barley stripe mosaic virus (BSMV) has been described as an effective VIGS vehicle for the evaluation of genes involved in wheat and barley phytopathogenesis; however, these studies have been obscured by BSMV-induced phenotypes and defense responses. The utility of BSMV VIGS may be improved using a BSMV genetic background which is more tolerable to the host plant especially upon secondary infection of highly aggressive, necrotrophic pathogens such as *Fusarium graminearum*.

**Results:**

BSMV-induced VIGS in *Triticum aestivum* (bread wheat) cv. ‘Fielder’ was assessed for the study of wheat genes putatively related to Fusarium Head Blight (FHB), the necrotrophism of wheat and other cereals by *F. graminearum*. Due to the lack of ‘Fielder’ spike viability and increased accumulation of *Fusarium*-derived deoxynivalenol contamination upon co-infection of BSMV and FHB, an attenuated BSMV construct was generated by the addition of a glycine-rich, C-terminal peptide to the BSMV γ b protein. This attenuated BSMV effectively silenced target wheat genes while limiting disease severity, deoxynivalenol contamination, and yield loss upon *Fusarium* co-infection compared to the original BSMV construct. The attenuated BSMV-infected tissue exhibited reduced abscisic, jasmonic, and salicylic acid defense phytohormone accumulation upon secondary *Fusarium* infection. Finally, the attenuated BSMV was used to investigate the role of the salicylic acid-responsive *pathogenesis*-*related 1* in response to FHB.

**Conclusions:**

The use of an attenuated BSMV may be advantageous in characterizing wheat genes involved in phytopathogenesis, including *Fusarium* necrotrophism, where minimal viral background effects on defense are required. Additionally, the attenuated BSMV elicits reduced defense hormone accumulation, suggesting that this genotype may have applications for the investigation of phytohormone-related signaling, developmental responses, and pathogen defense.

**Electronic supplementary material:**

The online version of this article (doi:10.1186/s13007-016-0112-z) contains supplementary material, which is available to authorized users.

## Background

Post-transcriptional gene silencing has been achieved in a variety of monocot and dicot plant species using virus-induced gene silencing (VIGS; for reviews see [1, 2]). This reverse genetic approach exploits a host plant’s endogenous RNA defense mechanism which recognizes the accumulation of foreign dsRNA and targets these sequences for degradation [3]. In this technique, viruses are engineered to encode sequences derived from the host plant transcriptome. Upon viral infection, both the viral genome and non-viral inserts are incorporated into a RNA-induced silencing complex, cleaved into interfering RNA molecules of 21–25 nt lengths, and used to degrade transcripts with sufficient sequence complementarity [4].

The barley stripe mosaic virus (BSMV) was identified as the first system to induce VIGS in monocot species [5]. BSMV is a positive sense RNA virus composed of a tripartite genome with its α genome encoding a RNA-dependent RNA polymerase methyltransferase/helicase subunit, the β genome encoding a viral coat protein and three triple gene block proteins, and γ genome encoding a RNA polymerase subunit and a γ b gene [6, 7]. Yuan and colleagues [8] described the stable incorporation of VIGS target sequences in the BSMV γ genome, directly after the γ b gene, using ligation independent cloning (LIC).

BSMV VIGS has been shown to be an effective tool in the functional genomics characterization of monocot genes involved in fungal pathogenesis including leaf and stem rust, powdery mildew, and wheat blast [9–11]. However, these studies have been complicated by the phenotypic and defense responses induced upon BSMV infection alone [1, 9, 10]. Additionally, pathogens investigated have been limited to biotrophic or hemibiotrophic lifestyles. One of the more costly cereal diseases is Fusarium head blight (FHB), the necrotrophic infection of wheat and other small grain cereals by species including *Fusarium graminearum* (for recent reviews see [12, 13]). This disease is especially devastating as it not only reduces grain yield but results in deoxynivalenol (DON) and other mycotoxin contamination of infected tissues. Upon FHB infection, wheat and barley elicit temporally-regulated salicylic (SA) and jasmonic acid (JA) defense responses [14–16]. Additionally, abscisic acid (ABA) and auxin (IAA) metabolite applications has been shown to increase and decreases FHB disease symptoms in susceptible cultivars, respectively [16, 17].

Due to its agronomic importance, robust virulence during necrotrophic infection, and characterized phytohormone defense responses, *F. graminearum* was selected as a secondary pathogen for VIGS of putatively related wheat genes. This work describes an attenuated BSMV genetic background for improved VIGS characterization of wheat genes involved in phytopathogenesis. This attenuated virus is capable of eliciting wheat gene silencing responses and is well tolerated upon BSMV/*Fusarium* co-infection. This increased tolerance reduced phytohormone defense responses, deoxynivalenol contamination, and yield loss. Therefore, modulation of BSMV infection severity may be useful for functional genomic characterization of monocot genes involved in pathogen infection or defense where minimal VIGS background is desirable.

## Results

**Fig. 1:**
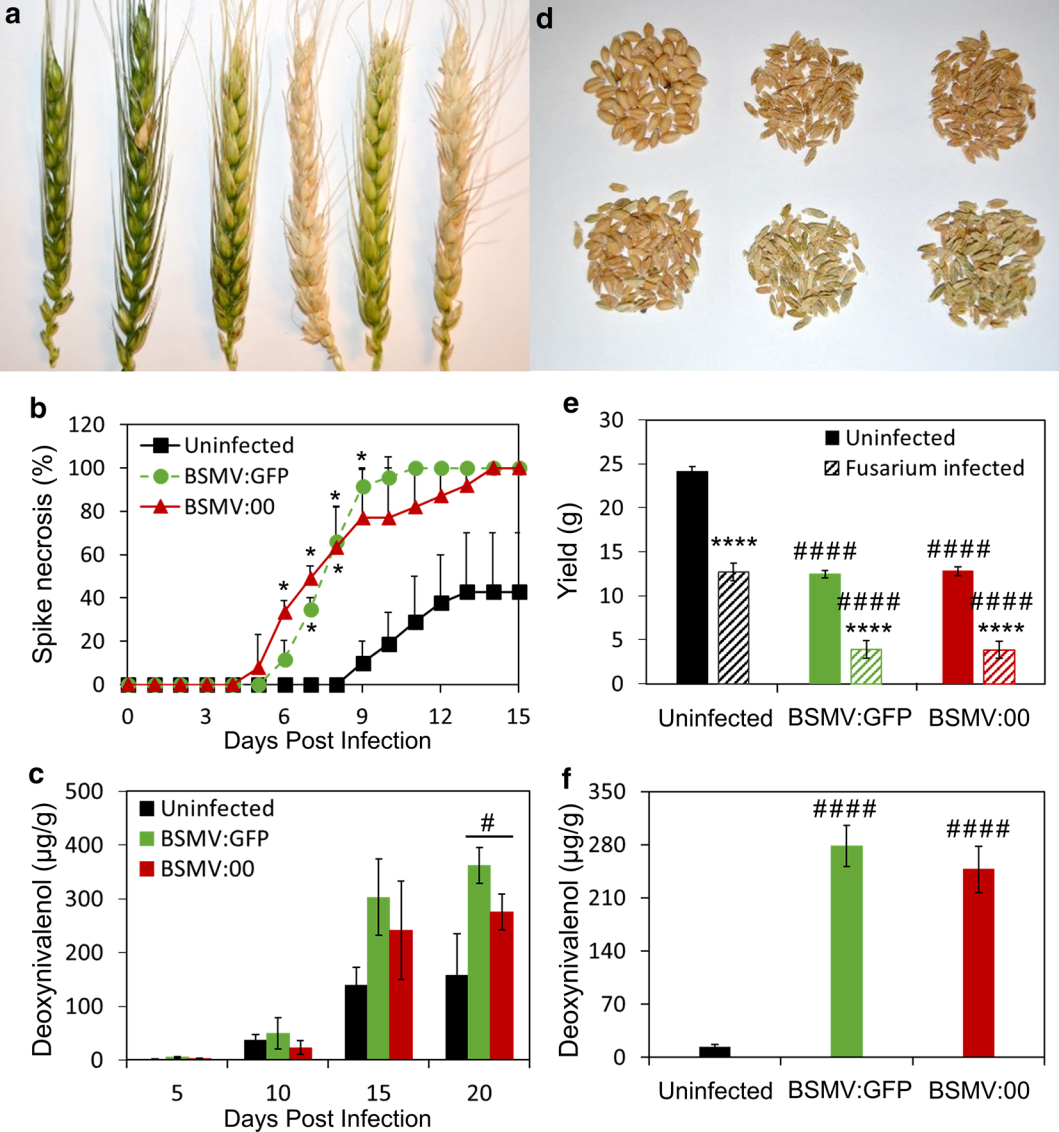
BSMV and *Fusarium* co-infection result in nonviable ‘Fielder’ spikes, increased DON contamination, and reduced yield. **a** WT, *Fusarium*-infected, BSMV:GFP-infected, BSMV:GFP and *Fusarium* co-infected, BSMV:00-infected, and BSMV:00 and *Fusarium* co-infected ‘Fielder’ spikes (from *left* to *right*) at 10 days post *Fusarium* inoculation. **b** Necrotic ‘Fielder’ spikes are averages of three replicate experiments, each composed of a minimum of 25 spikes, with standard error. **c** From one representative experiment, DON contamination was determined in five biological replicate spikes with standard deviation at 5 day intervals over the course of the infection. **d** Grains from non-BSMV, BSMV:GFP, and BSMV:00 infected tissue (*left* to *right*) without (*top*) and with (*bottom*) secondary *Fusarium* infection. **e** Yield represents the average of three biological replicate grain samples, sufficient for thousand grain weight calculation, from a representative experiment with standard deviation. ‘Uninfected’ on the x-axis refers to BSMV. ‘Uninfected’ in the inserted legend refers to *Fusarium*. **f** From one representative experiment, DON contamination was evaluated in three biological replicate grain samples with standard deviation. ‘Uninfected’ on the x-axis refers to BSMV. Differences in spike necrosis and DON contamination were analyzed with one-way ANOVA with Dunnett post hoc comparisons; while yield differences were evaluated with two-way ANOVA with Sidak post hoc comparisons. Changes between upon BSMV-infection are denoted with ‘#’ while *Fusarium* infected are denoted with ‘*’ (*p ≤ 0.05; ***p ≤ 0.001; ****p ≤ 0.0001)

To establish the utility of VIGS for FHB studies, the FHB-susceptible *Triticum aestivum* (bread wheat) cv. Fielder was infected during booting with BSMV encoding no VIGS-targeted gene fragment (BSMV:00) or BSMV encoding a 200 bp non-VIGS-targeted GFP fragment (BSMV:GFP). The presence of the BSMV infection alone created mosaic, bleached phenotypic symptoms on wheat spikes (for example Fig. 1a, third and fifth spike from left) beginning as early as 7 days after mechanical BSMV inoculation. During anthesis, a central spikelet on each spike was secondarily infected with purified *F. graminearum* spores. FHB can be phenotypically observed as a brown, necrosed spikelet at the site of spore application (for example Fig. 1a, second spike from the left) followed by necrosis of adjacent spikelets and the rachis as FHB spreads. This FHB spread can affect entire spikes, observed as brown, dried tissue (for example see Fig. 1a, fourth and sixth spikes from the left). The co-infection of BSMV and FHB resulted in a dramatic increase in spike necrosis and *Fusarium*–derived DON contamination in ‘Fielder’ spikes (Fig. 1a–c). This drastic response to co-infection was also observed in isolated grain which exhibited shriveled phenotypes, decreased yield, and increased DON contamination (Fig. 1d–f). Comparison between the BSMV constructs, BSMV:00 and BSMV:GFP, resulted in no significant differences in ‘Fielder’ spike viability, grain yield, or DON contamination.

Characterization of ‘Fielder’ spikes suggest that BSMV/*Fusarium* co-infection may be too severe for FHB-susceptible cultivars and may therefore be a less than desirable approach for functional genomic identification of wheat genes involved in FHB. One potential strategy to overcome this limitation may be to modulate BSMV infection severity while maintaining gene silencing. BSMV packaging and assembly, movement, pathogenesis, and post-transcriptional gene silencing have all been shown to involve γ *b* [7, 18, 19]. Deletion of this gene results in reduced accumulation of the BSMV RNA and coat (βa) and βb proteins ultimately preventing the vascular movement of the virus in select host plants [8, 18, 19]. Functional characterization of the γ b protein identified two N-terminal cysteine-rich, zinc finger-like domains separated by a basic linker region and a C-terminal six heptad coiled-coil motif [20, 21]. Mutational studies of this C-terminal coiled-coil motif support its role in homo-oligomer formation, BSMV virulence, and BSMV counter-defenses in monocot and dicot hosts [21].

**Fig. 2:**
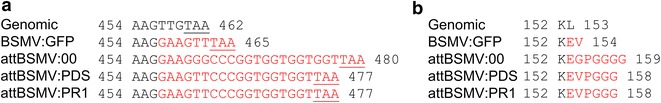
Sequence alignment of BSMV γ b constructs. **a** Gene and **b** protein sequence alignments with red nucleic and amino acids demonstrating the 3′ and C-terminal differences with underlined stop codons

Interestingly, the method proposed for high throughput, monocot gene silencing using BSMV by Yuan et al. [8] relied on cloning gene fragments of interest into a genomically stable site directly 3′ to the BSMV γ *b* gene. In an attempt to modulate BSMV severity by altering the coiled-coil formation of γ b, a BSMV construct with an additional C-terminal peptide was generated by uncontrolled T4 DNA polymerase base excision at the LIC site. This construct lacked the native γ *b* stop codon, causing an additional seven-mer peptide of Glu-Gly-Pro-Gly-Gly-Gly-Gly encoded on the C-terminus of the protein (Fig. 2). Due to its ability to lessen BSMV symptoms, improve viability upon secondary *Fusarium* infection, and reduce phytohormone defense responses (described in the following paragraphs), this BSMV construct will henceforth be referred to as the attenuated BSMV without a VIGS insert (attBSMV:00). Further research is required to understand how this glycine-rich C-terminal tail affects the functions of γ b as well as BSMV assembly, movement, and post-transcriptional gene silencing.

**Fig. 3:**
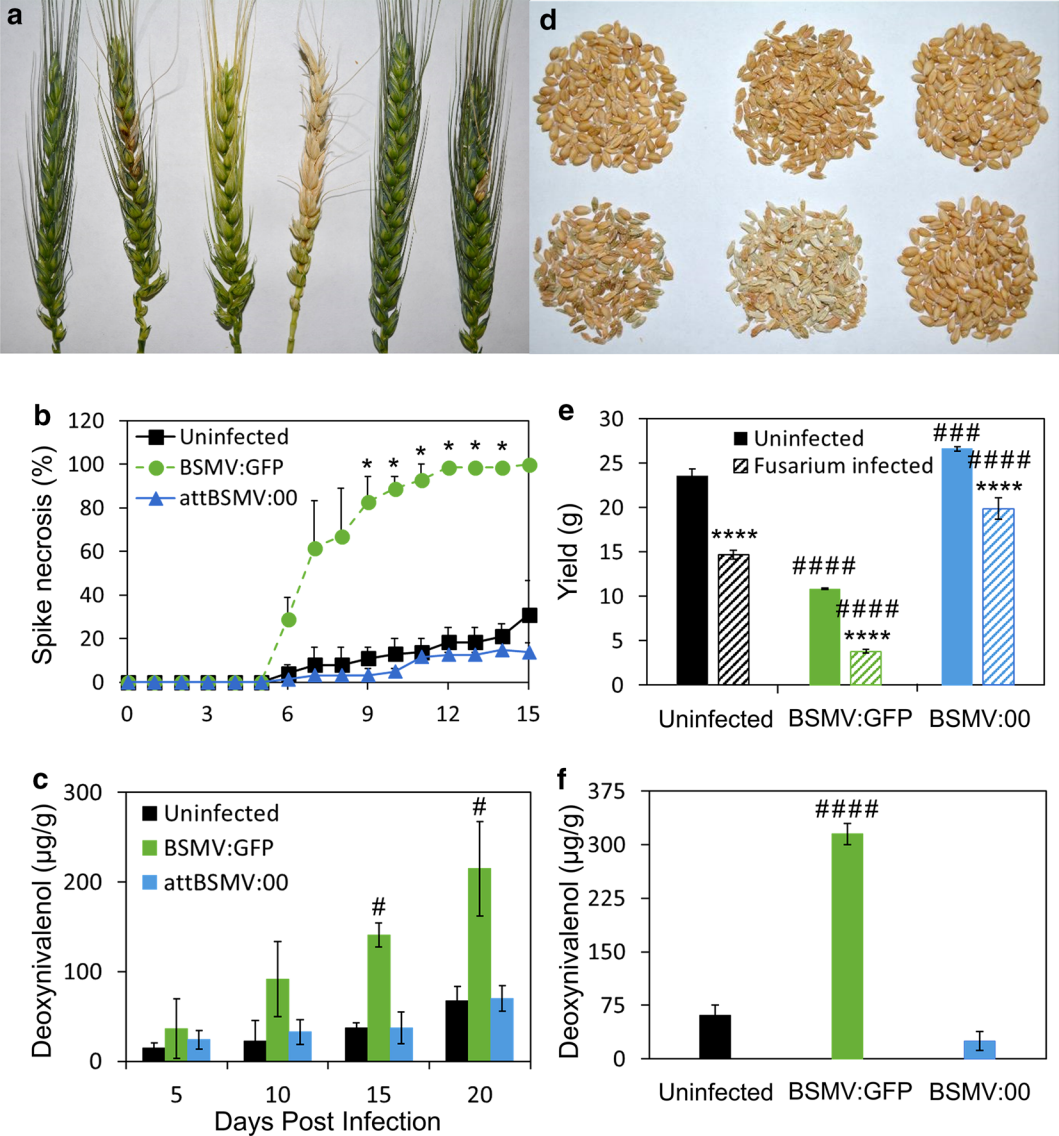
attBSMV:00 exhibits improved *Fusarium* co-infection tolerance, reduced DON contamination, and increased yield compared to BSMV:GFP-infected ‘Fielder’ tissue. **a** WT, *Fusarium*-infected, BSMV:GFP-infected, BSMV:GFP and *Fusarium* co-infected, attBSMV:00-infected, and attBSMV:00 and *Fusarium* co-infected ‘Fielder’ spikes (*left* to *right*) at 10 days post *Fusarium* inoculation. **b** Necrotic ‘Fielder’ spikes are averages of three replicate experiments, each composed of a minimum of 25 spikes, with standard error. **c** From one representative experiment, DON contamination was determined in five biological replicate spikes with standard deviation at 5 day intervals over the course of the infection. **d** Grains from non-BSMV, BSMV:GFP, and attBSMV:00 infected tissue (*left* to *right*) without (*top*) and with (*bottom*) secondary *Fusarium* infection. **e** Yield represents the average of three biological replicate grain samples, sufficient for thousand grain weight calculation, from a representative experiment with standard deviation. ‘Uninfected’ on the x-axis refers to BSMV. ‘Uninfected’ in the inserted legend refers to *Fusarium*. **f** From one representative experiment, DON contamination was evaluated in three biological replicate grain samples with standard deviation. ‘Uninfected’ on the x-axis refers to BSMV. Differences in spike necrosis and DON contamination were analyzed with one-way ANOVA with Dunnett post hoc comparisons; while yield differences were evaluated with two-way ANOVA with Sidak post hoc comparisons. Changes between upon BSMV-infection are denoted with ‘#’ while *Fusarium* infected are denoted with ‘*’ (*p ≤ 0.05; ***p ≤ 0.001; ****p ≤ 0.0001)

To determine if attBSMV:00 may be a practical alternative for functional genomic characterization of wheat genes compared to the BSMV:GFP, FHB was evaluated in combination with each viral genotype. ‘Fielder’ spikes were infected with BSMV:GFP or attBSMV:00 and were secondarily infected during anthesis with purified *F. graminearum* spores. ‘Fielder’ spikes infected with attBSMV:00 exhibited reduced BSMV-induced phenotypic bleaching, necrosis upon *Fusarium* co-infection, and DON contamination compared to spikes infected with BSMV:GFP (Fig. 3a–c). This improved tolerance upon attBSMV:00/*Fusarium* co-infection was also observed in isolated grain which exhibited lesser phenotypic shriveling, no yield decrease, and limited DON contamination (Fig. 1d–f). Ultimately, the comparison of these two viral genotypes suggests that the attBSMV:00 may be a more appropriate system to investigate the role of wheat genes on FHB pathogenesis. The comparison of these constructs also highlights a caveat of BSMV VIGS, namely that the effect of VIGS targeted knockdown is a combined effect of a particular gene’s influence on FHB severity and also the less apparent BSMV infection potency.

In addition to improving wheat viability upon BSMV*/Fusarium* co-infection, attBSMV:00-infected tissue exhibited reduced accumulation of defense phytohormones compared to the BSMV:GFP-infected tissue. Previous characterization of salicylic (SA) and jasmonic (JA) acid biosynthetic genes and metabolite applications suggest both hormones play a role in defense against *Fusarium* infection [14–16]. ABA has been shown to modulate other disease responses and is therefore also considered along with its catabolites dihydrophaseic and phaseic acids [17, 22–25]. To characterize phytohormone defense responses upon BSMV infection, including those induced with a secondary *Fusarium* infection, phytohormone content was quantified in ‘Fielder’ spikes infected with BSMV:GFP or attBSMV:00. During late-stage infection (21 days after mechanical inoculation), conjugated SA was reduced by BSMV:GFP infection and attBSMV:00-infection by 41 and 63 % respectively (Table 1). At this same infection stage (14 days after *Fusarium* inoculation and 21 days after BSMV inoculation), BSMV:GPF and *Fusarium* co-infection increased ABA levels two-fold, dihydrophaseic acid levels 13-fold, phaseic acid levels threefold, JA levels eightfold, JA-isoleucine levels almost ninefold, and SA levels sixfold in comparison to the *Fusarium*-only infected tissue. In contrast, attBSMV:00 and *Fusarium* co-infected tissue only reduced conjugated SA 14-fold compared to the *Fusarium*-only infected tissue. These hormone profiles of ‘Fielder’ spikes infected with BSMV:GFP or attBSMV:00 suggests that attBSMV:00 may be advantageous for the functional genomic characterization of wheat genes involved in defense responses or phytohormone-regulated metabolism.

**Table 1:** Phytohormone profiles are less affected by the attBSMV:00 compared to BSMV:GFP infection

	Uninfected (ng/g DW)	*Fusarium* infected (ng/g DW)
ABA		
WT	154 ± 31.0	227 ± 62.7
BSMV:GFP	193 ± 29.5	458 ± 54.2^##,^ *
attBSMV:00	144 ± 23.8	346 ± 122*
Dihydrophaseic acid		
WT	28.2 ± 37.4	71.4 ± 64.7
BSMV:GFP	20.5 ± 7.8	916 ± 96.7^####, ^****
attBSMV:00	31.1 ± 7.2	52.7 ± 19.8
Phaseic acid		
WT	112.5 ± 9.3	213 ± 98.4
BSMV:GFP	84.0 ± 38.7	678 ± 353^#,^ **
attBSMV:00	65.8 ± 28.1	83.4 ± 22.0

	Uninfected (ng/g WW)	*F. graminearum* (ng/g WW)
JA		
WT	22.8 ± 3.4	63.7 ± 12.1
BSMV:GFP	27.1 ± 7.9	506.0 ± 105.6^####, ^****
attBSMV:00	15.8 ± 3.8	40.8 ± 6.8
JA-Isoleucine		
WT	10.9 ± 4.6	20.3 ± 3.9
BSMV:GFP	7.6 ± 2.1	174.9 ± 31.4 ^####,^ ****
attBSMV:00	8.4 ± 2.8	16.0 ± 6.9
SA		
WT	121.8 ± 32.3	137.1 ± 56.4
BSMV:GFP	260.6 ± 67.6	835.3 ± 152.4^####,^ ****
attBSMV:00	96.2 ± 23.6	91.8 ± 27.9
Conjugated SA		
WT	1121.9 ± 77.0	681.7 ± 49.0**
BSMV:GFP	657.8 ± 246.2^##^	786.1 ± 65.3
attBSMV:00	415.5 ± 86.8^####^	228.3 ± 16.2^##^

Values represent the average of three biological ‘Fielder’ spike replicates with standard deviation. ABA and its catabolites, dihydrophaseic and phaseic acids, were quantified from lyophilized tissue [dry weight (DW)]; while JA, SA, and metabolites were quantified without a lyophilization step [wet weight (WW)]. Differences upon BSMV (denoted by ‘#’) or *Fusarium* infection (denoted with ‘*’) were evaluated by two-way ANOVA with Sidak post hoc comparisons (*p ≤ 0.05; **p ≤ 0.01; ***p ≤ 0.001; ****p ≤ 0.0001)

The functional genomic application of attBSMV:00 is dependent on the assembled virus’s ability to infect ‘Fielder’ spike tissue and induce post-transcriptional gene silencing. To assess the ability of attBSMV to induce VIGS knockdown, a 200 bp fragment of the *phytoene desaturase* (PDS) was LIC cloned into BSMV γ with additional DNA encoding a glycine-rich, C-terminal peptide on γ b (Additional file 1: Table S1; Fig. 2). PDS has been used as a VIGS phenotypic biomarker in wheat leaf and spike tissue as silencing increases chlorophyll photobleaching resulting in chlorosed tissue [5, 26]. The generation of the attBSMV construct encoding a PDS fragment was problematic as the original attBSMV:00 construct was generated by unregulated T4 polymerase base excision that eliminated the LIC site. To maintain the LIC site for efficient VIGS fragment cloning, a BSMV construct with a modified glycine-rich, C-terminal extension of BSMV γ b was generated (attBSMV:PDS, Fig. 2). This secondary peptide retained the original LIC construct valine [8] and encoded one fewer C-terminal glycine residue compared to attBSMV:00.

**Fig. 4:**
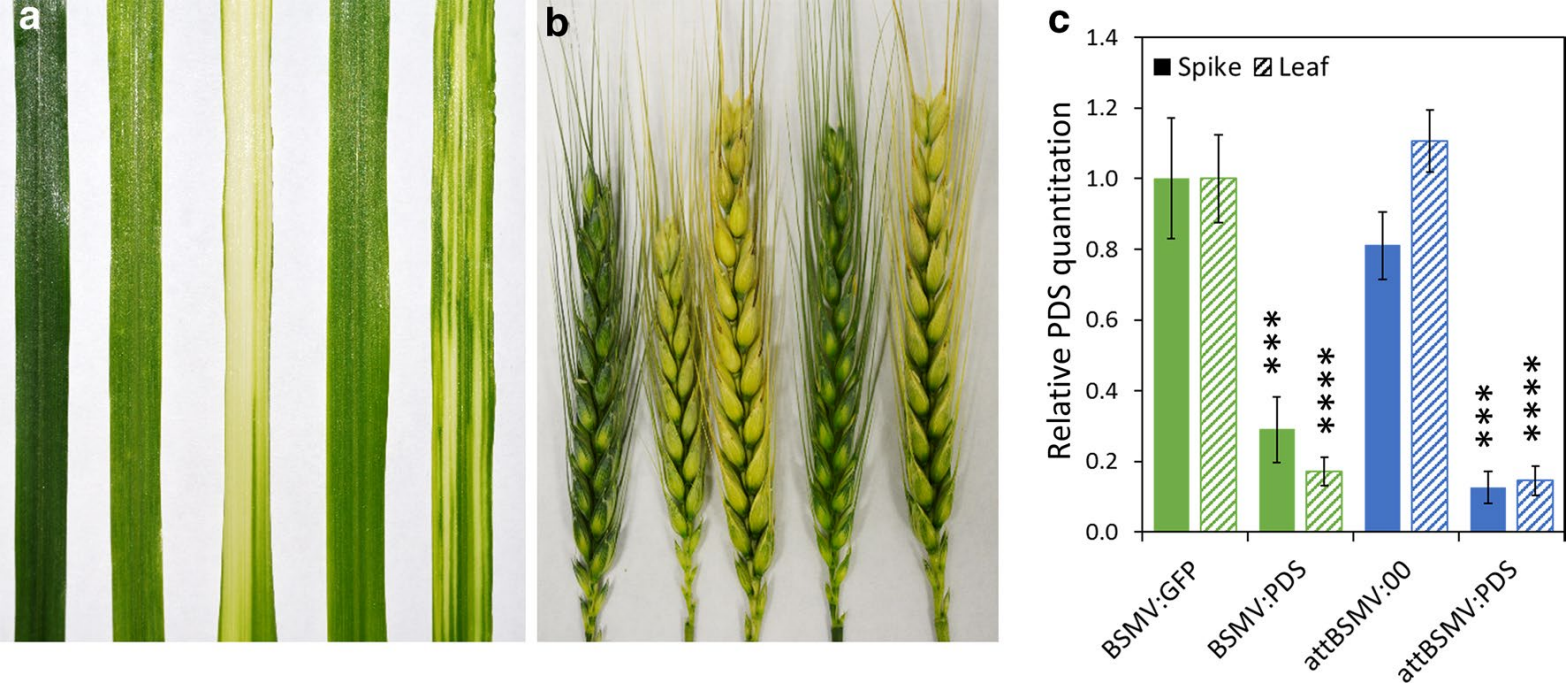
attBSMV:00 is capable of VIGS in ‘Fielder’ leaves and spikes. Uninfected, BSMV:GFP-infected, BSMV:PDS-infected, attBSMV:00-infected, and attBSMV:PDS-infected (*left* to *right*) ‘Fielder’ **a** leaves and **b** spikes at 15 days post BSVM inoculation. **c** Relative PDS quantitation by semiquantitative RT-PCR represents the average of three biological replicate ‘Fielder’ leaves or spikes at 15 days post BSMV inoculation, from a representative experiment with standard deviation. Differences were evaluated with two-way ANOVA with Sidak post hoc comparisons (*p ≤ 0.05)

To assess the ability of the attBSMV:PDS construct to induce VIGS knockdown, ‘Fielder’ leaves and spikes were mechanically inoculated, monitored for tissue photobleaching, and evaluated for relative PDS gene expression. To control for viral infection and PDS-dependent photobleaching, the BSMV:GFP and BSMV:PDS positive control infections in leaves and spikes were performed in parallel. ‘Fielder’ leaf and spike tissue infected with attBSMV:PDS exhibited photobleaching as early as 15 days post inoculation and reduced *PDS* gene expression by approximately 80 % in both tissue types (Fig. 4). Although the attBSMV is capable of migrating from the mechanical inoculation sites to characterized leaf and spike tissue and eliciting gene silencing, functional and physiological characterization of the attBSMV compared to the BSMV genotype may be of substantial interest to describe changes in phenotypic penetrance, viral mobility, virulence, and gene silencing upon the addition of the γ b glycine-rich, C-terminal peptide.

**Fig. 5:**
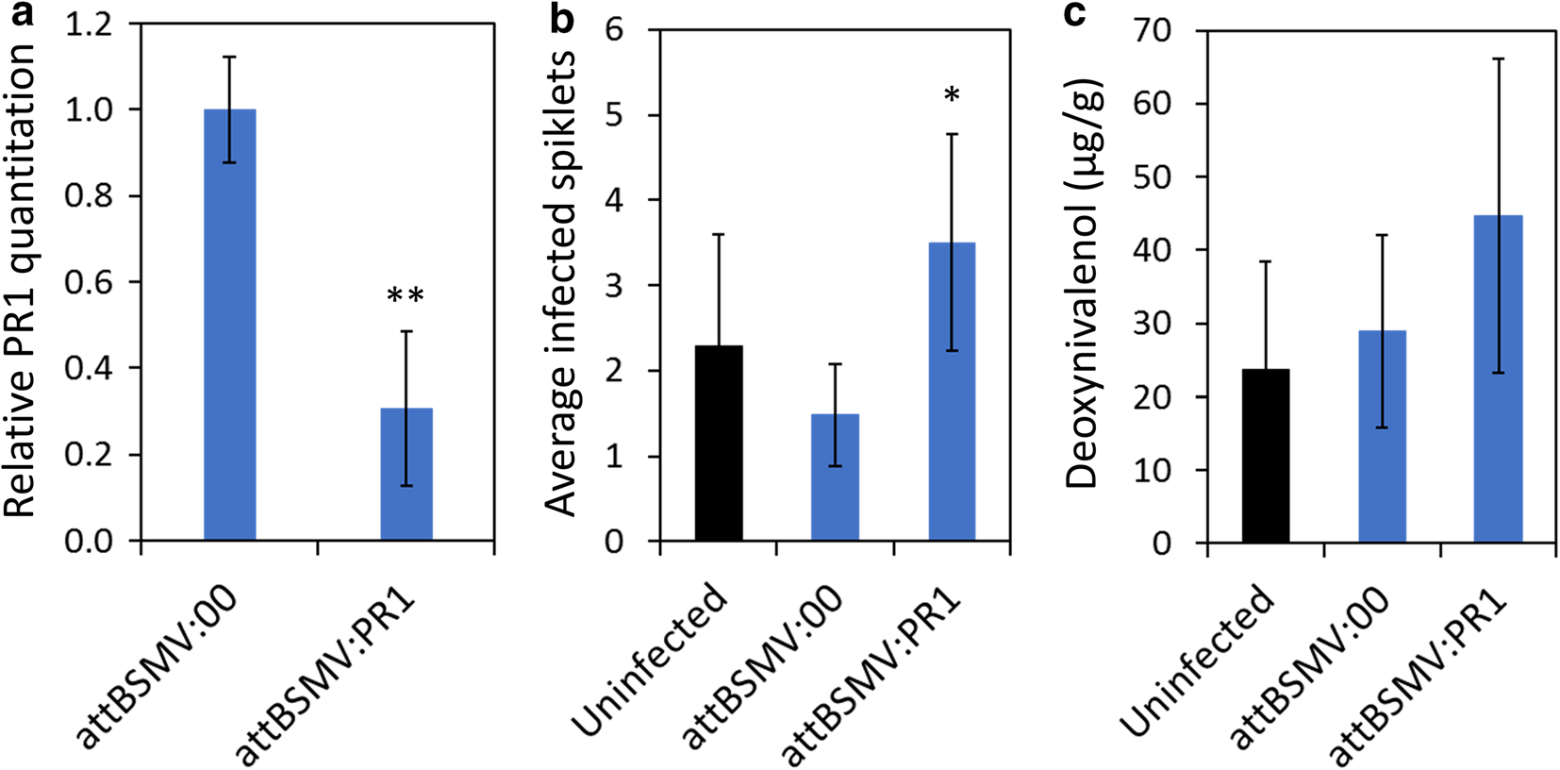
attBSMV:00 may be used to characterize the role of wheat genes in FHB pathogenesis. **a** Relative PR1 quantitation values represent the average of three biological replicates ‘Fielder’ spikes at 7 days post BSMV inoculation with standard deviation. **b** FHB-infected spikelets were calculated as the average of three independent experiments, each composed of a minimum of 25 spikes, with standard error. **c** DON was detection in ten biological replicates from a representative experiment with deviation. Differences between the attBSMV:00 and attBSMV:PDS are evaluated with T-tests (*p ≤ 0.05)

The reduced pathogenic severity and effective VIGS exhibited by the attBSMV constructs suggest this viral genotype may present a more suitable approach for the functional genomic identification of wheat genes involved in FHB pathogenesis. To more fully establish this utility, the effect of *pathogenesis*-*related 1* (PR1) on *Fusarium* infection was evaluated. PR family members are involved with SA defense signaling upon pathogen infection (reviewed in [27, 28]) and have been confirmed in *F. graminearum* basal resistance [28]. Indeed, *Fusarium*-infected wheat demonstrate increased PR1 expression [29, 30]; while wheat lines overexpressing PR1 exhibit stronger SA accumulation and defense than non-transgenic plants [14]. As such, a 200 bp gene fragment of PR1 was LIC cloned into BSMV γ with the attBSMV:PDS peptide addition (Additional file 1: Table S1; Fig. 2). Infection of this PR1 VIGS-targeted construct (attBSMV:PR1) produced a moderate phenotypic bleaching in ‘Fielder’ spikes (Additional file 1: Figure S1) accompanied by the relative reduction of PR1 gene expression by approximately 70 % compared to attBSMV:00 construct at 7 days post inoculation (Fig. 5a). This data provides a second example, to that of PDS (Fig. 4), of the effectiveness of the attBSMV at mediating gene knockdown in wheat.

In terms of FHB impact, unlike ‘Fielder’ spikes infected with BSMV lacking the γ b C-terminal peptide (Figs. 1, 2), tissue infected with either the attBSMV:00 or attBSMV:PR1 remained viable at and beyond 10 days post *Fusarium* infection (Additional file 1: Figure S1). This highlights that the minor variations in the sequence of the glycine rich tag, attached at the γ b C-terminus in the PDS and PR1 constructs (Fig. 2), do not significantly alter the attenuating effect, compared to that originally observed for the attBSMV:00 glycine rich γ b C-terminal peptide (Fig. 1). Furthermore, attBSMV:PR1-infected ‘Fielder’ spikes exhibited increased *Fusarium* spread and a slight, although not significant, increase in DON contamination compared to attBSMV:00 (Fig. 5b, c). However, it is important to note that due to the minor differences in the attBSMV:00 and attBSMV:PR1, these results do not address the BSMV-induced background phenotype but highlights the importance of optimizing BSMV constructs to appropriately modulate viral attenuation in a manner that is appropriate for a secondary pathogen or defense response of interest. Thus, while suggesting the possibility of a role for PR1 in plant responses to FHB and BSMV infection, further characterization of this gene is required for conclusive evidence. Overall, the characterization of the attBSMV genotype suggests this system may be applied to effectively target the post-transcriptional knockdown of wheat genes in leaf and spike tissue such that the tissue remains viable during secondary *Fusarium* infection.

## Discussion

FHB is a devastating disease of wheat and other small grain cereals that reduces agronomic yield and grain quality and has been reported throughout Europe, Asia, Africa, North America, and Australia (For recent reviews see [12, 13]). There are no commercially-available, highly FHB-resistant wheat cultivars, and disease management has been limited to the application of fungicides (for example [31–33]) or biological control agents (for example [34–36]). To develop wheat varieties with improved FHB resistance, plant breeding efforts have been aimed at identifying FHB resistant QTLs (for example [37–40]). These wheat breeding efforts, as well as the identification of novel fungicide drug targets, may be greatly assisted by functional genomic characterization of putative FHB-related genes using the VIGS construct system described herein as a basis.

The BSMV construct identified in this work demonstrated reduced immune responses as evidenced by reduced phytohormone accumulation, and thus could be applied to a number of emerging problems in wheat and barley research. For example, knowledge of phytohormone signaling and cross-talk in monocot species could be expanded to characterize mechanisms dictating appropriate development and environmental responses [41]. Additionally, current research efforts have been focused on the improvement of wheat tolerance to extreme temperatures, drought stress, and reduced fertilizer applications [42–44]. As these investigations are dependent on applying abiotic stressors, VIGS systems which mitigate genotype stress responses may provide a better system for characterizing associated responsive genes.

The application of BSMV VIGS is not without caveats. Ramanna et al. [45] recently outlined a number of problematic areas including BSMV’s inability to infect select cultivars, low penetrance, transient phenotype, severe and obscuring phenotypic symptoms, and induction of plant defense response genes. Optimization of BSMV constructs and protocols to address specific experimental requirements, such as genetic manipulation of the BSMV γ *b* gene described in this work, may be required prior to high throughput functional genomic screening of monocot genes by BSMV VIGS.

## Conclusions

Functional genomic characterization of wheat genes involved in plant development and pathogen defense have previously been described using BSMV VIGS. However, this system has been reported to generate strong phenotypic symptoms and induce wheat defense responses. In this work, an attenuated viral construct was created by the addition of a glycine-rich, C-terminal peptide to BSMV γ b. This construct exhibited reduced BSMV phenotypic symptoms while generating robust VIGS of wheat PDS and PR1. Upon *Fusarium* co-infection, this attBSMV also exhibited increased ‘Fielder’ spike viability and reduced ABA, JA, and SA phytohormone defense responses. BSMV VIGS is an emerging technology that may be further developed for the characterization of genes involved in phytopathogenesis by modulating viral pathogenesis.

## Methods

### Ligation independent cloning of barley stripe mosaic virus constructs

LIC cloning was performed as described in [8] using the Barley Stripe Mosaic α, β, γ (or derivatives) generously provided by Dawei Li, State Key Laboratory of Agro-Biotechnology at China Agriculture University. For the LIC cloning of VIGS targeting γ constructs: *T. aestivum* PDS [GenBank: FJ517553.1] and PR1 [GenBank: HQ848391] gene fragments were amplified with gene-specific primers flanked with LIC complementary sequences and sequences to encode the γ b C-terminal, glycine-rich peptide (Additional file 1: Table S1). All primers in this work were designed using Primer3 v0.4.0 [46]. PCR amplicons were purified using the QIAquick PCR Purification Kit (Qiagen, Mississauga, CA), treated with T4 DNA polymerase (New England Biolabs, MA, USA) in 1× reaction buffer containing 5 mM dATP for 20 min, and heat-treated at 75 °C for 10 min to inactivate the polymerase. The BSMV γ plasmid was linearized by *Apa*I (New England Biolabs) digestion, extracted in phenol:chloroform, and treated with T4 DNA polymerase as above with the exception of the presence of 5 mM dTTP instead of dATP. The T4 DNA polymerase-treated PCR products and BSMV γ plasmid vector were mixed, incubated for 2 min at 66 °C and allowed to anneal as the mixture cooled to room temperature over 30 min. Finally, 2 µl aliquots were transformed into high efficiency *Escherichia coli* DH5α (Life Technologies) per the manufacturer instructions and sequence-confirmed by Sanger DNA sequencing at NRC-Saskatoon. The BSMV γ plasmids were transformed into *Agrobacterium tumefaciens* C51 for subsequent agroinfiltration. In the case of attBSMV:00, the exact mechanism of construct generation is unknown, as it was originally identified by sequencing as a mis-cloning product.

### Virus-induced gene silencing in ‘Fielder’ leaves and spikes

*Agrobacterium tumefaciens* harboring BSMV α, β, and γ plasmids were propagated in 5.0 ml LB media supplemented with 25 µg/ml rifampicin and 100 µg/ml kanamycin at 25 °C overnight. Cells were pelleted and resuspended to an OD_600_ of 0.7 in agrofiltration buffer (100 mM 2-(*N*-morpholino) ethanesulfonic acid pH 5.2, 10 mM MgCl_2_, 0.1 mM acetosyringone). Equal volumes of α, β, γ encoding a gene fragment from the Tomato Bushy Stunt Virus P19 for the promotion of gene silencing [47, 48], and γ encoding a VIGS targeting gene fragment-harboring cells were combined, incubated at room temperature for 3 h, and infiltrated into 4 week old *Nicotiana benthamiana* using sterile, needless syringes. Infiltrated *N. benthamiana* was grown for 7 days in climate controlled chambers under 16:8 h, 25:16 °C day: night cycles. Infiltrated leaves were harvested and ground in 20 mM sodium phosphate buffer pH 7.0, 40–100 mesh silicate (BHD Inc; Toronto, ON), and 200–450 mesh silicon carbonate (Sigma-Aldrich; St. Louis, MO). The resulting *N. benthamiana* mixture was mechanically inoculated into *T. aestivum* cv. ‘Fielder’ plants (grown in identical climate controlled chamber conditions as described for *N. benthamiana*) at the two-leaf stage or on the flag leaves during booting just prior to spike emergence. VIGS treatments were performed on a minimum of 25 ‘Fielder’ spikes per treatment.

To confirm VIGS knockdown, total RNA was extracted from ‘Fielder’ fourth-emerging leaves or individual spikelets using the RNeasy Plant Mini Kit (Qiagen, Mississauga, CA) and treated with *DNaseI* (Qiagen, Mississauga, CA) according to the manufacture’s instruction. cDNA was synthesized using 0.5 µg RNA and the Superscript III reverse transcriptase kit (Invitrogen, Carlsbad, CA) according to the manufacture’s instruction. Target gene knockdown was confirmed based on gene expression relative to the *T. aestivum* heterogeneous nuclear ribonucleoprotein Q (*hn*-*PNP*-*Q*, Unigene: Ta.10105 [15]) reference gene by semi-quantitative RT-PCR. Comparative C_T_ (ΔΔC_T_ method [49]) was applied using the StepOne Plus Real-Time PCR System (Applied Biosystems, Foster City, CA) and the associated StepOne Software v2.3 (Thermo Fisher Scientific Inc., Carlsbad, CA). For VIGS of *T. aestivum* PDS, leaf and spike tissue were observed until photobeaching was apparent prior to RNA isolation at 15 days post infection; while for VIGS of wheat PR1, RNA was isolated immediately prior to *F. graminearum* inoculation at 7 days post infection.

### Fusarium head blight infection

*Fusarium graminearum GZ3639* (generously provided by Susan McCormick at the United State Department of Agriculture [50]) was sequentially propagated from on potato dextrose agar (Sigma-Aldrich; St. Louis, MO) for 5 days and in carboxymethylcellulose liquid media (CMC; Sigma-Aldrich; St. Louis, MO) for 7 days. Spores were isolated by filtering CMC culture through one layer of cheesecloth and one layer of 25 μm Miracloth filter (EMD Millipore; Billerica, MA), washed three times with sterile water, and counted using light microscopy. During anthesis, 7 days after BSMV infection, two florets from a central spikelet of ‘Fielder’ were inoculated with 10 µl 5.0 × 10^4^*F. graminearum* spore suspension or deionized water (mock). *F. gramineaum* inoculations were performed on a minimum of 25 spikes per treatment. Wheat plants were then transferred to 90 % humidity conditions for 72 h.

### Wheat hormone quantification

Three biological replicate ‘Fielder’ spikes inoculated with each treatment were flash frozen and ground in liquid nitrogen. Treatments include uninfected, BSMV:GFP infected, attBSMV:00 infected, *Fusarium* infected, BSMV:GFP and *Fusarium* co-infected, and attBSMV:00 and *Fusarium* co-infected. Phytohormones were extracted and quantified by UPLC/ESI–MS/MS at NRC-Saskatoon as described in [51–55].

### Yield and DON quantification

Thousand seed weight, a measure of yield, was determined as five times the mass of three groups of 200 randomly selected seeds as described [56]. To determine DON contamination, three or five biological replicates of 1.0 g of pooled *‘*Fielder’ grains or individual spikes, respectively, were ground to a fine powder in liquid nitrogen. DON was extracted in five volumes of 84 % (v/v) acetonitrile by shaking at 220 rpm, 25 °C for 2 h. DON was quantified, relative to a commercial DON standard (Sigma-Aldrich; St. Louis, MO), by LC–MS through modification of [57]. Briefly, DON was separated with a Waters 2695 LC coupled with a Waters Symmetry C18 column (100 × 2.1 mm ID, 3.5 µm) with mobile phases of 0.3 % (v/v) acetic acid (A) and 95 % (v/v) methanol: 0.3 % (v/v) acetic acid (B) using a gradient elusion from 0 to 7 min: 99 % A, 7 to 25 min: 67 % A 33 % B, and 25 to 30 min 99 % A. The flow rate and column temperature were maintained at 0.2 ml/min and 25° C, respectively. Mass spectrum analysis was performed using a Waters 3100 Mass Detector fitted with ESI in negative ion mode and an optimized 40 V cone voltage. DON was detected at *m/z* of 355.3 Da and analyzed using Empower Pro Software (Waters, Milford, MA).

### Statistical analysis

Single dependent variable comparisons with single treatments (PR1 expression, *Fusarium* spikelet infection, and DON contamination) were analyzed with T-tests. Single dependent variable comparisons with multiple treatments (DON detection) were analyzed with one-way ANOVA with Dunnett post hoc comparisons. Multiple dependent variable comparisons (yield, hormone quantification, and detection of PDS expression) were analyzed with two-way ANOVA with Sidak post hoc comparisons. All comparisons were performed with GraphPad Prism 6 (GraphPad Software, Inc. La Jolla, CA).

## Additional file

10.1186/s13007-016-0112-z Table S1. LIC cloning and semi-quantitative RT-PCR primers; and Figure S1. FHB phenotype in ‘Fielder’ spikes.

## Abbreviations

ABA: abscisic acid
BSMV: barley stripe mosaic virus
BSMV:00: barley stripe mosaic virus with no insert
BSMV:GFP: barley stripe mosaic virus encoding a GFP gene fragment
BSMV:PDS: barley stripe mosaic virus encoding a wheat PDS gene fragment
attBSMV:00: barley stripe mosaic virus with C-terminal peptide but no insert
attBSMV:PDS: barley stripe mosaic virus with C-terminal peptide and PDS gene insert
attBSMV:PR1: barley stripe mosaic virus with C-terminal peptide and PR1 gene insert
DON: deoxynivalenol
FHB: Fusarium head blight
JA: jasmonic acid
LIC: ligation independent cloning
PR1: pathogenesis-related 1
PDS: phytoene desaturase
SA: salicylic acid
VIGS: virus-induced gene silencing
WT: wildtype
