# Supplementary material for: Identification of an attenuated barley stripe mosaic virus for the virus-induced gene silencing of pathogenesis-related wheat genes
Source: Plant Methods. 2016 Feb 2;12:12. doi: 10.1186/s13007-016-0112-z (PMC4736275; doi:10.1186/s13007-016-0112-z)
Supplement: Supplementary file 1 — 10.1186/s13007-016-0112-z Table S1. LIC cloning and semi-quantitative RT-PCR primers; and Figure S1. FHB phenotype in ‘Fielder’ spikes. [file 13007_2016_112_MOESM1_ESM.docx]

**Identification of an attenuated Barley Stripe Mosaic Virus for the virus-induce gene silencing of putative Fusarium Head Blight-related wheat genes**

Leann M. Buhrow^1^, Shawn M. Clark^1^ and Michele C. Loewen^1,2*^

1. Aquatic and Crop Research Development Portfolio, National Research Council Canada, 110 Gymnasium Place, Saskatoon, SK S7N 0W9 Canada

2. Department of Biochemistry, University of Saskatchewan, 107 Wiggins Rd. Saskatoon, SK., S7N 5E5 Canada

**Supplemental Material**

**Table S1: LIC cloning and semi-quantitative RT-PCR primers.** BSMV constructs were amplified using primers containing the flanking LIC adaptors (in red) and sequence to encode the glycine-rich C-terminal γ b extension (in blue). VIGS knockdown was confirmed by relative gene expression using semi-quantitative RT-PCR using gene specific primers compared to the *T. aestivum* heterogeneous nuclear ribonucleoprotein Q (*hn-PNP-Q*, Ta.10105 [1]) reference gene using the comparative C_T_ (ΔΔC_T_ method [2]). Primer efficiency and standard correlation are noted for RT-PCR primers only; while BSMV cloning primers are noted ‘not applicable’ (NA).

| qPCR VIGS  silencing | | | LIC cloning  constructs | | **Method** |
| --- | --- | --- | --- | --- | --- |
| PR1 | PDS | *hn-RNP-Q* | attBSMV:PR1 | attBSMV:PDS | **Gene** |
| GCGGGAATATCATTGGACAG | GCATGGAAGGATGAAGATGGTG | TCACCTTCGCCAAGCTCAGAACTA | **AAGGAAGTTCCCGGTGGTGGTT**AACCAAGCTAGCCATCTTGCTC | **AAGGAAGTTCCCGGTGGTGGTTAA**TTTCTCCAGGAGAAG | **Forward Primer** |
| TGCGATTAGGGACGAAAGAC | GAGTGTTCCTTCCATTGCAAGC | agttgaacttgcccgaaacatgcc | **AACCACCACCACCGT**GAGCTTGCAGTGTTGATCC | **AACCACCACCACCGT**TCTCCAGTTATTTGAG | **Reverse Primer** |
| 93.7 | 95.5 | 92.4 | NA | NA | **Efficiency** |
| 0.994 | 0.961 | 0.945 | NA | NA | **Correlation** |

**Figure S1: FHB phenotype in ‘Fielder’ spikes.** (A) WT, (B) attBSMV:00-infected, and (C) attBSMV:PR1-infected ‘Fielder’ spikes at ten days post *Fusarium* inoculation.

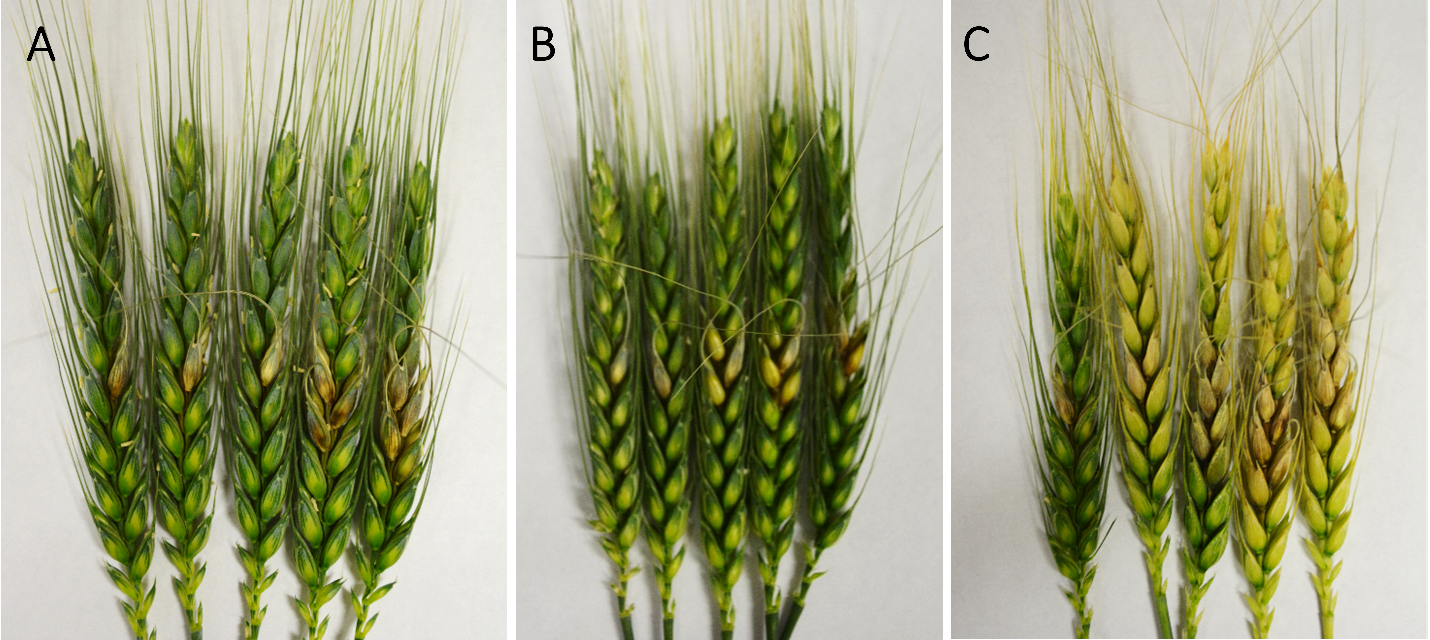


**Supplemental References:**

1. Qi PF, Johnson A, Balcerzak M, Rocheleau H, Harris LJ, Long XY, Wei YM, Zheng YL, Ouellet T. Effect of salicylic acid on Fusarium graminearum, the major causal agent of fusarium head blight in wheat. Fungal Biology 2012; 116(3):413-426.

2. Livak KJ, Schmittgen TD. Analysis of relative gene expression data using real- time quantitative PCR and the 2^-ΔΔCT^ method. Methods 2001; 25:402-408.

3. Yuan C, Li C, Yan L, Jackson AO, Liu Z, Han C, Yu J, Li D. A high throughput Barley Stripe Mosaic Virus vector for virus induced gene silencing in monocots and dicots. PLoS ONE 2011; 6(10):e26468.
